# The Different Contributors to Antioxidant Activity in Thermally Dried Flesh and Peel of Astringent Persimmon Fruit

**DOI:** 10.3390/antiox11030597

**Published:** 2022-03-21

**Authors:** You Jin Lim, Seok Hyun Eom

**Affiliations:** Department of Smart Farm Science, College of Life Sciences, Kyung Hee University, Yongin 17104, Korea; yujn0213@khu.ac.kr

**Keywords:** thermal drying, antioxidants, carotenoids, condensed tannins, hydrolysable tannins

## Abstract

In the thermal-drying processing of astringent persimmon fruit, the tissue-specific changes in the key antioxidants have hardly been investigated, while they have been well investigated in the flesh. We report here the different patterns of the antioxidant activities in the thermally processed flesh and peel of astringent persimmon, with analyses of the carotenoids, the condensed and hydrolysable tannins, and the total phenolics and flavonoids. The persimmon powders presented different colors on the basis of the drying temperatures: brown in 30 °C; light yellow in 60 °C; and dark brown in 90 °C, respectively. Non-maillard reaction and reduction of carotenoids caused the light-yellow color of 60 °C dried persimmon. Thermal drying reduced the antioxidant activities of the flesh in a temperature-dependent manner, with decreases in the carotenoids, the condensed and hydrolysable tannins, and the total phenolics and flavonoids, whereas it enhanced the antioxidant activities of the peel. The increase in the antioxidant activities in the peel were mainly the result of the increase in the total phenolics by the thermal effect, and especially in the content of the hydrolysable tannins, although the thermal processing decreased the other components. The heat-induced increase of antioxidant activity in the peel showed a strong significant correlation only with the contents of total phenolics (*r*^2^ = 0.9493) and total hydrolysable tannins (*r*^2^ = 0.9288), suggesting that the main antioxidant contributors differ from the flesh.

## 1. Introduction

Persimmons (*Diospyros* L.) are classified into astringent and nonastringent cultivars on the basis of their astringency levels. Astringent persimmon fruits have astringency indexes that are about 15 times higher than those of nonastringent persimmons, while there is little difference in the total soluble solid (18.3–29.5°Brix, depending on the cultivar) and fresh weight (61–216 g) [1]. Although the astringent persimmon fruit is a rich source of bioactive compounds with antioxidant activity, such as phenolics, flavonoids, carotenoids, and tannins [2,3], it is less preferred because of its astringent taste and undesirable flavor. Fruits generally undergo thermal processing to enhance their sensorial and preservative qualities. During persimmon fruit processing, the fruit peel is usually produced as a byproduct, which is disposed of as an industrial waste. Most commercial products of persimmon, such as juice, sherbet, or puree, are produced from the peeled persimmon flesh [2]. Nevertheless, it has been reported that persimmon peel exhibits physiological activities, such as antioxidant, antigenotoxic, anti-atherogenic, and tyrosinase inhibition [4,5]. In addition, active compounds, including myricitrin, the glycoside of quercetin and kaempferol, and 2-methoxy-4-vinylphenol, have been isolated from persimmon peel [4,6,7]. Drying is one of the most commonly used processing methods for fruit preservation and further processing. Hot-air drying is popular and is a widely used drying method because of its easy operation and low cost [8,9]. It has been reported that the temperature of hot-air drying has a significant effect on the antioxidants and the antioxidant activity of persimmon flesh, whereas there are few reports on the thermal changes of the antioxidants and their activities in persimmon peel.

Persimmon fruit has been extensively studied in the food and pharmaceutical industries as a potential nutraceutical fruit with health and medical benefits, including the prevention of cardiovascular disease and diabetes, and the alleviation of hangover symptoms [10]. Chemical changes generally occur during the processing of persimmon fruit. Previous studies report the thermal or dehydration-based changes in carotenoids and tannins, which are the major active compounds in persimmon flesh. For example, dehydration and heating can induce the isomerization of trans- to cis-carotenoids [11]. Although carotenoids are well-known antioxidants, a recent report shows that heat drying the persimmon flesh does not affect their content, but that it does lower the antioxidant activity, which suggests structural changes of the carotenoids during processing [11]. The flesh of persimmon fruit predominantly contains highly polymerized condensed tannins as the major antioxidants [12]. Highly polymerized tannins are structurally unlinked and can be degraded by heat [13]. “Condensed” and “hydrolysable” are the two major classes of tannins; however, the latter is rarely reported in persimmon. During persimmon dehydration, extractable condensed and hydrolysable (gallic acid and tannic acid) tannins are converted into nonextractable tannins [14]. Most of the previous studies on persimmon tannins have focused on reducing their astringency or on quantification techniques.

Unlike the industrially available information on persimmon flesh, the peel is considered a byproduct, and therefore, the studies characterizing the carotenoids and tannins in persimmon peel remain limited. In addition, the analysis of the antioxidant properties in persimmon peel has been limited to studies that compare the activities of astringent and nonastringent peels [15], that evaluate the antioxidant and antigenotoxic activities of solvent-extracted peels [5,16], as well as assays that attempt to increase the antioxidant activities of peels by heat treatment [17]. Similarly, the carotenoid content has only been comparatively quantified in persimmon peel and flesh [18]. As the ratio of the carotenoid composition varies in the flesh and peel of persimmon [19], the changes in the carotenoid content that are due to heating could also differ, depending on the persimmon part that is being analyzed and the heating temperature. Several flavonoids from persimmon peel, such as myricitrin, kaempferol, and quercetin, have been studied as potential antioxidants [6,7]. However, an increase in the antioxidant activity of peels after heat treatment, but with no significant change in the contents of the antioxidant compounds, has been observed. Interestingly, despite a previous report that 2-methoxy-4-vinylphenol is the major antioxidant in persimmon peel [4], it is an aromatic compound that could easily be volatilized by heat drying. In contrast, few studies on the quantification of the tannins in the peels of persimmon have been reported. The correlation between the phytochemicals and the antioxidant activity under heat and the dehydration factors during the processing of persimmon is not well established. The ability of thermal drying to significantly affect the antioxidant properties of persimmon fruit can potentially be used to establish the relationship between the antioxidant activity and the contents of carotenoids and tannins. Understanding carotenoids and tannins as major antioxidants in persimmon fruit, and the changes in their contents during dehydration and heating, is important for the development of functional materials in the food industry.

Here, we aimed: (1) To investigate the changes in the carotenoids, the condensed tannins, and the hydrolysable tannins in astringent persimmon fruit under varying heat intensities; and (2) To analyze the major antioxidant contributors in the flesh and peel of persimmon fruit. For this purpose, the carotenoid content, the total condensed and hydrolysable tannins, and the total phenolic and flavonoid contents in the flesh and peels of astringent persimmon fruit were analyzed after being subjected to thermal drying under four different temperature regimes. In addition, the antioxidant activities were evaluated, and a correlation analysis between the phytochemicals and the antioxidant activities was performed.

## 2. Materials and Methods

### 2.1. Fruit Sampling and Thermal Processing

Astringent persimmon (*Diospyros kaki* Thunb. cv. Cheongdo-Bansi) fruit was harvested on 19 October 2020 in Cheongdo (35°35′19.6″ N; 128°44′54.8″ E), which is located in South Korea. A total of 10 fruits per tree were harvested from 10 trees. The harvested fruits were characterized as 173.8 ± 9.1 g of fresh weight; 25.7 ± 1.1 N/m^2^ of hardness; and 16.0 ± 0.5°brix of the total soluble solid, on average. The harvested fruits were stored at 1 °C and 90% relative humidity for 2 weeks before being used in the experiment. The peeled flesh was sliced to a thickness of about 3 mm, which refers to a previous report for the sufficient dehydration and heat transfer [20]. The peel was completely separated from the flesh by peeling to a thickness of 1 mm. The sliced flesh and peel were dried with different temperatures: freeze-drying for 72 h in a vacuum freeze dryer (IlshinBioBase Co. Ltd., Dongducheon, Korea); and hot-air drying at 30, 60, and 90 °C for 72 h, respectively. After the thermal treatment, the moisture was completely removed in a 30 °C dry oven (KED-066A, Kiturami, Seoul, Korea). Triplicate samples were ground using a commercial grinder (BL142, Tefal, Rumilly, France). Each sample (6 g) was extracted with 180 mL of 80% ethanol (*v/v* for 24 h at 25 °C, and 120 rpm in a shaking incubator (DS-210SF, Daewonsci Inc., Bucheon, Korea)), as per a previous report, with some modification [21]. The extract was concentrated using a vacuum rotary evaporator (Eyela Co., Tokyo, Japan) at 38 °C to prevent chemical changes after filtration by using the Whatman No. 2 filter paper (Whatman International Ltd., Maidstone, UK). The concentrated extracts were dissolved in 80% ethanol (100 mg/mL) and were used for further analysis.

### 2.2. Colorimetric Assays

Sample images were taken using a DSLR camera (D5000, Nikon, Tokyo, Japan) with a resolution of 12.3 megapixels. The camera was placed vertically on a 60 cm-high stand. The distance between the lens and the sample was constant. For the uniform light intensity (20 μmol/m^2^/s) without shadows on the sample, the fluorescent lamps were located at a 45° angle and 50 cm above the samples. The color parameters of the ground samples were obtained in the RGB system by using a color analyzer (Lutron Electronics, Inc., Coopersburg, PA, USA). The RGB values were converted into L* (luminosity), a* (green-to-red), and b* (blue-to-yellow) CIELab coordinates by using the OpenRGB v. 2.30.10125. software (Logicol, Trieste, Italy). The color difference (Δ*E*) was calculated as follows [22]: Δ*E* = (ΔL*^2^ + Δa*^2^ + Δb*^2^)^1/2^. The browning values of the Maillard reaction products (MRPs) were measured as was previously described [22]. For the determination of the browning values, the absorbance of 80% of the crude ethanol extracts (20 mg/mL) was measured at 420 nm by using a spectrophotometer (S-4100; SCINCO Co., Ltd., Seoul, South Korea).

### 2.3. Carotenoid Measurement Using HPLC

The saponification was performed before the determination of the carotenoids by using a previously described method with modifications [18]. Methanol (350 µL) was added to the dried and ground samples (50 mg). After the mixture was vortexed for 1 min, 700 µL of chloroform was added and vortexed. Subsequently, 10% NaCl (350 µL) was added and mixed homogeneously. The chloroform phase (bottom layer) was collected after centrifugation (15 °C; 8000× *g*; 5 min). Diethyl ether (200 µL) was added to the chloroform phase and mixed. Then, 1 N KOH (350 µL) was added, mixed, and incubated in a heating block (DW-110, Daeil Tech Co., Ltd.) at 60 °C for 30 min under light blocking. Subsequently, 10% NaCl (350 µL) was added and mixed homogeneously. The mixture was centrifuged at 15 °C and 8000× *g* for 5 min. The chloroform phase was collected and mixed with 700 µL of 10% NaCl to wash the KOH. The mixture was centrifuged at 15 °C and 8000× *g* for 5 min. Then, the chloroform phase was collected and filtered through a 0.45 µm syringe filter. The excess solvent was removed by using a vacuum rotary evaporator (Eyela Co.) at 38 °C. The concentrated extracts were dissolved in 800 µL of ethyl acetate and were loaded to a reversed-phase HPLC (Waters 2695 Alliance HPLC, Waters, Milford, MA, USA) after filtration (0.45 µm hydrophilic PTFE syringe filter, Futecs Co., Ltd., Daejeon, South Korea).

The HPLC determination of the carotenoids was performed by gradient elution on a C18 column (Prontosil 120-5-C18SH-EPS 5 µm (250 × 4.6 mm; Bischoff, Leonberg, Germany)) with a Waters 2695 Alliance HPLC (Waters Inc., Milford, MA, USA), in accordance with the previously published method [21]. The mobile phase was a combination of Solvent A (water/acetonitrile (10:90, *v*/*v*) with 0.1% formic acid) and Solvent B (ethyl acetate with 0.1% formic acid). The gradient elution was performed as follows: 0–60% of Solvent B for 12 min; and 60–65% of Solvent B for 23 min, and then re-equilibration to the initial gradient. The flow rate was 1 mL/min. The injection volume was 10 µL. The peaks were monitored at 450 nm with a Waters 996 photodiode array detector (Waters Corporation, Milford, MA, USA). The zeaxanthin and β-carotene were identified by using standard compounds (Extrasynthase, Genay, France). The identification of the β-cryptoxanthin and carotenoid esters was performed according to the elution order in the C18 column and their UV-vis spectra on the basis of a previous report [23].

### 2.4. Measurement of Hydrolysable Tannins Using HPLC

Gallic and tannic acids were analyzed using a Kinetex 5 μm C18-100A column (150 × 4.6 mm; Phenomenex, Torrance, CA, USA), which is described by previous methods, with some modifications [12,24]. The flow rate of the mobile phase was 1.0 mL/min. The injection volume was 10 µL. The mobile phase was a combination of Solvent A (water with 0.4% formic acid) and Solvent B (acetonitrile): 5% Solvent B for 5 min; 5–80% Solvent B for 5–10 min; 80–90% Solvent B for 10–15 min; and 90% Solvent B maintained for 2 min. The eluents were monitored at 280 nm. The gallic and tannic acids were purchased for the HPLC standards (Sigma Aldrich Co., St Louis, MO, USA).

### 2.5. Assays of Total Condensed Tannins (TCT) and Total Proanthocyanidins (TPA)

The TCT content was measured by a vanillin method with some modifications [25]. The samples (400 µL; 10 mg/mL) were mixed with 3 mL of 4% vanillin in methanol and 1.5 mL of 37% HCl. After 15 min of incubation at 22 °C, the absorbance was measured at 500 nm. Catechin was used as a standard for the quantification. The TPA content was measured by an *n*-butanol–HCl method with some modifications [3]. The mixing ratios of the sample and the butanol–HCl solution were modified. The butanol–HCl solution was prepared by mixing *n*-butanol and 12 N HCl with 95:5 (*v/v*), and it contained 0.37 mM of Fe_2_(SO_4_)_3_·H_2_O. The samples (300 µL; 10 mg/mL) were mixed with 900 µL of butanol–HCl solution. The mixtures were incubated at 95 °C for 30 min in a heating block (DW-110, Daeil Tech Co., Ltd., Seoul, Korea). After incubation, the mixtures were cooled on ice and centrifuged at 4 °C and 10,000× *g* for 3 min. The absorbance was measured at 550 nm. The absorbances of the nonheated mixtures of each treatment were measured as blanks. The TPA content was calculated as follows:

Total proanthocyanidins = (ODsample − ODblank) × 0.1736 ^a^

^a^ 0.1736 is the conversion factor that is calculated from a previous report [3].

### 2.6. Assays of Total Hydrolysable Tannins (THT)

The THT contents were determined by KIO_3_ and FeCl_3_ assays, with some modifications [26,27]. The mixing ratios of the samples and the solutions of KIO_3_ and FeCl_3_ were modified. The reaction time was also modified. For the KIO_3_ assay, 400 µL of the extract (10 mg/mL) was mixed with 800 µL of 2.5% KIO_3_. After 4 min of incubation at 22 °C in the dark, the mixtures were centrifuged (10,000× *g*; 3 min; 4 °C). The absorbance of the supernatant was measured at 550 nm. For the FeCl_3_ assay, 950 µL of the extract (10 mg/mL) was mixed with 50 µL of 1% methanolic FeCl_3_. After 15 min of incubation with the same conditions that were mentioned above, the absorbance was measured at 530 nm. Gallic and tannic acids were used as the standards in the KIO_3_ and FeCl_3_ assays, respectively.

### 2.7. Measurement of Total Phenolics (TPs) and Flavonoids (TFs), and Antioxidant Activities

The TP and TF contents were determined as previously described [28]. The contents were expressed as mg gallic acid equivalents (GAE) and catechin equivalents (CE)/g dry weight (D.W.) of the plant material, respectively. The antioxidant activities, the 2,2-diphenyl-1-picrylhydrazyl (DPPH), the 2,2′-azino-bis (3-ethylbenzothiazoline-6-sulphonic acid) (ABTS), and the ferric-reducing ability of plasma (FRAP) were evaluated by a previously described method [28]. The activities were expressed as mg vitamin C equivalents (VCE)/g D.W.

### 2.8. Statistical Analysis

All data are expressed as the mean ± standard error of the three replicate experiments. All statistical analyses were performed using the SAS software (Enterprise guide, Version 7.1; SAS Institute Inc., Cary, NC, USA). The significant differences among the thermal processing in these experiments were evaluated by using Tukey’s studentized range test at a significance level of *p* < 0.05. In all the figures and tables, the different letters next to the values indicate a significant difference. The correlation analysis was performed by calculating the Pearson’s correlation coefficients between the phytochemicals and the antioxidant activities with SAS software.

## 3. Results

### 3.1. Effect of the Drying Temperature on Color Attributes

**Figure 1:**
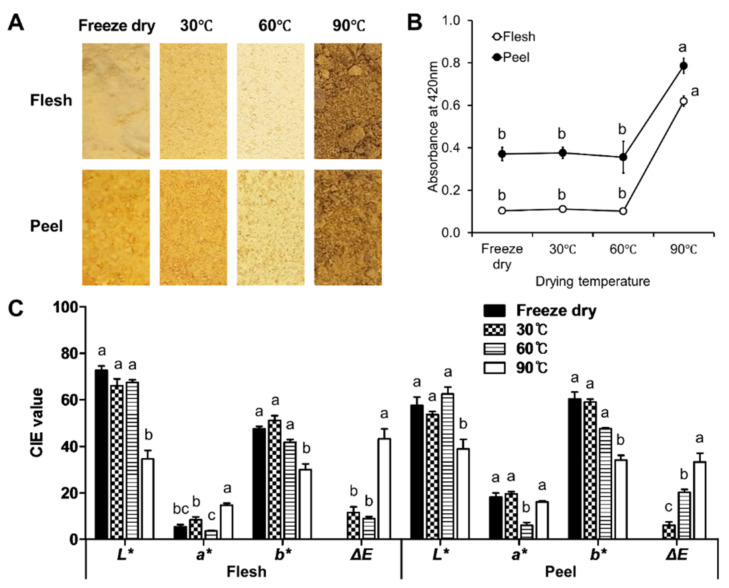
Visual color changes of astringent persimmon fruit (coarsely ground powder) dried at different temperatures: (**A**) browning value of the MRPs (Maillard reaction products) by absorbance at 420 nm; and (**B**) their CIE L*, a*, and b* values and color difference (Δ*E*); (**C**) different letters in each parameter of each sample of flesh and peel indicate significant differences at *p* < 0.05 (Tukey’s HSD test).

The browning values did not differ among the freeze-dried, and the 30 °C- and 60 °C-dried samples, and they presented averages of 0.11 and 0.38 of the absorbent values in the flesh and the peel, respectively. However, they significantly increased at 90 °C drying temperature, where they exhibited 0.62 in the flesh, and 0.79 in the peel, respectively (Figure 1). The CIELab colorimeter results show similar color patterns in the L* (luminosity) and b* (blue-to-yellow) values (Figure 1C). The L* and b* values were higher among the freeze-dried and the 30 °C- and 60 °C-dried treatments, and they did not significantly differ from each other, while the values were significantly low in the 90 °C-dried samples of both the flesh and the peel. These results indicate that the 90 °C-dried samples lost brightness and yellowness. Unlike the L* and the b* values, the a* (green-to-red) values showed different patterns among the thermally dried samples and between the persimmon tissues. The a* values of the flesh samples increased as the processing temperature increased, except for 60 °C. Otherwise, the a* values of the peel were not significantly different among the freeze-dried and the 30 °C- and 90 °C-dried peels. Similar to the flesh, the a* value of the 60 °C-dried peel was significantly lower than that of the other treatments. Interestingly, the 60 °C drying treatment caused both tissues to present as whitish yellow (Figure 1A). The color difference (Δ*E*) clearly exhibits the browning degree and the color loss among the thermal treatments. The Δ*E* value of the 90 °C-dried flesh was significantly higher than those of the others, and it exhibited a dark brown color.

### 3.2. Change in Carotenoids after Thermal Drying

**Figure 2:**
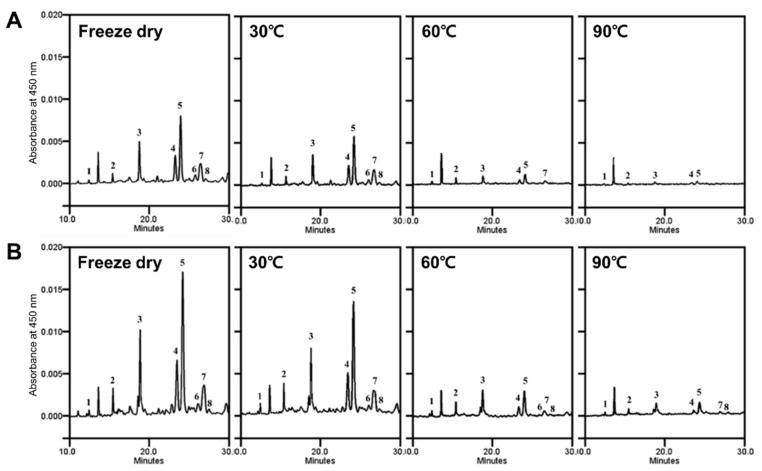
HPLC chromatograms of carotenoids in the flesh (**A**) and peel (**B**) extracts of astringent persimmon fruit dried at different temperatures. Each peak was identified as follows; 1, zeaxanthin; 2, β-cryptoxanthin; 3, β-carotene; 4, β-cryptoxanthin ester; 5, β-cryptoxanthin ester; 6, zeaxanthin ester; 7, zeaxanthin ester; 8, zeaxanthin ester.

Eight carotenoid peaks, including zeaxanthin, β-cryptoxanthin, β-carotene, two peaks of β-cryptoxanthin esters, and three peaks of zeaxanthin esters, were detected (Figure 2). The differences in the carotenoid compositions of the thermally dried flesh and peel are presented in Table 1. The content of the total carotenoids in the freeze-dried peel was 2.5-fold higher than that in the freeze-dried flesh. All eight carotenoids were higher in the peel than in the flesh, even during thermal drying. β-cryptoxanthin ester was a dominant carotenoid in the persimmon flesh and peel, followed by β-carotene, zeaxanthin ester, and another β-cryptoxanthin ester. All the carotenoids decreased after thermal drying in both the flesh and the peel. The total carotenoids in the flesh decreased by about 25, 86, and 95% in the 30, 60, and 90 °C treatments, respectively, compared to the freeze-dried sample. The total carotenoids in the peel decreased by about 32, 83, and 93% at 30, 60, and 90 °C, respectively. The β-carotene, β-cryptoxanthin esters, and zeaxanthin esters were drastically decreased at 60 °C, and to a more severe extent at 90 °C.

### 3.3. Changes in TCT and THT

**Figure 3:**
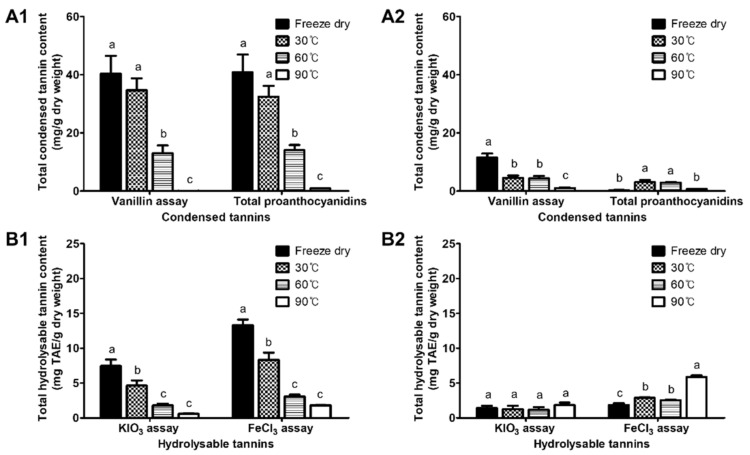
Total condensed tannin (**A1**,**A2**) and total hydrolysable tannin (**B1**,**B2**) contents of thermally dried flesh (**A1**,**B1**) and peel (**A2**,**B2**) of astringent persimmon fruit. Total condensed tannin content evaluated by vanillin assay was expressed as mg CE/g D.W. Total hydrolysable tannin contents were expressed as mg tannic acid equivalent (TAE)/g D.W. Different letters in each assay indicate significant differences at *p* < 0.05 (Tukey’s HSD test).

The freeze-dried flesh had the highest TCT (40.35 mg/g D.W.) and TPA (40.82 mg/g D.W.) contents among all of the drying treatments (Figure 3(A1)). The TCT and TPA contents in the flesh significantly decreased at 60 and 90 °C, and hence, the TCT and TPA contents were hardly detected at 90 °C, and they ranged between 0.01 and 0.95 mg/g D.W. In the peel, the TCT content decreased during thermal drying, whereas the TPA content slightly increased at 30 and 60 °C (Figure 3(A2)). The THT content in the flesh significantly decreased during the thermal drying in both assays (Figure 3(B1,B2)). Otherwise, the THT content in the peel during thermal drying showed a different pattern between the two assays: there was no change in the content by the KIO_3_ assay, whereas the content by the FeCl_3_ assay increased as the drying temperature increased.

### 3.4. Determination of Hydrolysable Tannins Using HPLC Analysis

**Figure 4:**
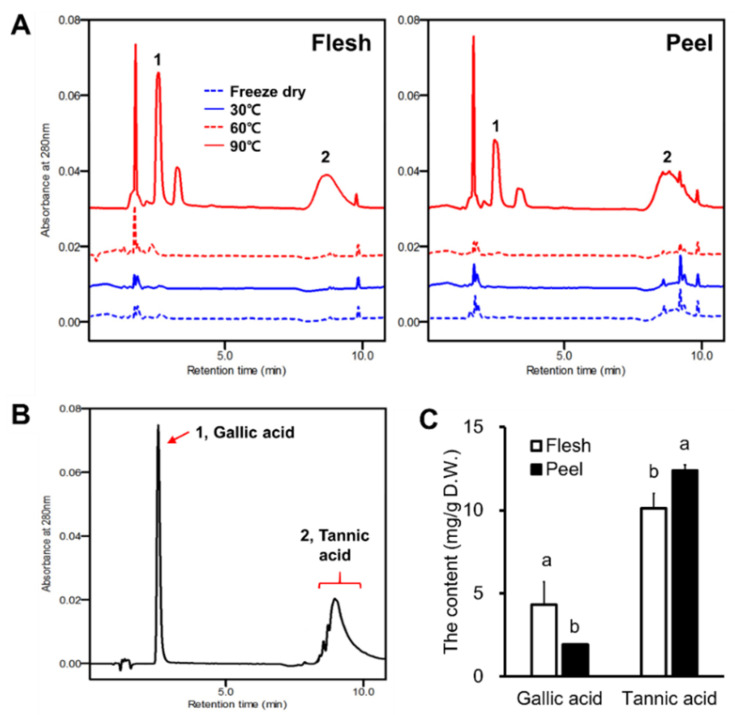
HPLC chromatograms of (**A**) the extracts of flesh and peel dried at different temperatures, (**B**) hydrolysable tannin standards (gallic and tannic acids), and (**C**) the content of gallic and tannic acids in the 90 °C dried flesh and peel. Different letters between the flesh and peel of each compound in Figure 4C indicate significant differences at *p* < 0.05 (Tukey’s HSD test).

Gallic acid was detected at a retention time of 2.42 min, while the standard of the tannic acid was detected at a retention time between 8 and 10 min, and as a broad peak that included several peaks within a hump (Figure 4B), which is consistent with a previous report [29]. Both the gallic and tannic acid contents in the flesh and peel were increased by thermal processing. Notably, the tannic acid hump was not observed in the flesh samples that were freeze-dried, nor in those that were dried at 30 and 60 °C. However, tannic acid was distinctly detected in the flesh and the peel that were dried at 90 °C (Figure 4A). In contrast, few peaks representing the tannic acid hump were detected in the freeze-dried peel and in those dried at 30 and 60 °C. Gallic acid was higher in the persimmon flesh than in the peel, with concentrations of 4.34 and 1.92 mg/g D.W., respectively. Interestingly, a 1.22-fold higher content of tannic acid in the peel than in flesh was observed in the samples dried at 90 °C (Figure 4C).

### 3.5. TP and TF Contents

**Figure 5:**
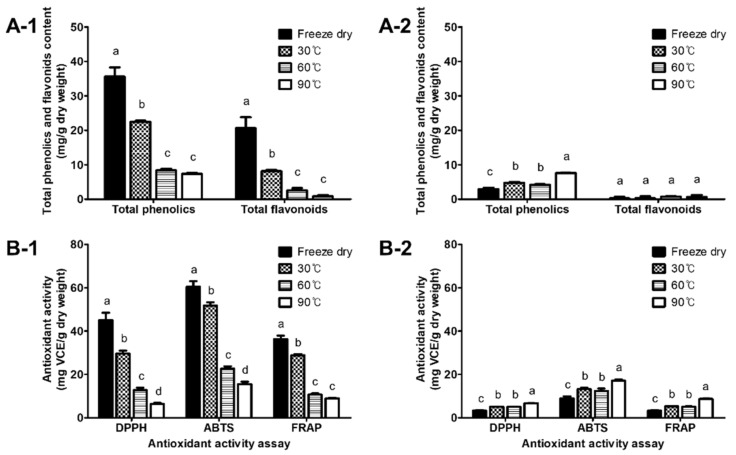
The total phenolic and flavonoid contents and (**A-1**,**A-2**) antioxidant activities; and (**B-1**,**B-2**) in thermally dried flesh (**A-1**,**B-1**) and peel (**A-2**,**B-2**) of astringent persimmon fruit. Total phenolics and flavonoids were expressed as mg GAE and CE/g D.W., respectively. Antioxidant activities were expressed as mg VCE/g D.W. Different letters in each assay indicate significant differences at *p* < 0.05 (Tukey’s HSD test).

The TP and TF contents of the freeze-dried samples were higher in the flesh than in the peel. Thermal drying significantly decreased the TP and TF contents in the flesh (Figure 5(A-1)). The TP contents gradually decreased with the increase in the temperature, and were decreased by 36.9 and 79.3% in the 30 °C- and 90 °C-dried flesh compared to the freeze-dried flesh. The TF contents were also decreased by 60.6 and 95.9% in the 30 °C- and 90 °C-dried flesh, respectively. In contrast to the flesh, the TP content in the peel gradually increased as the drying temperature increased. The TF content in the peel showed no significant changes during thermal drying.

### 3.6. Antioxidant Activities

In the freeze-dried samples, the flesh had much higher antioxidant activities, exhibiting 45.12, 60.48, and 36.83 mg VCE/g D.W. in the DPPH, ABTS, and FRAP, respectively, than the peel, which presented 3.31, 9.01, and 3.31 mg VCE/g D.W. in the assays (Figure 5(B-1,B-2)). There was a significant reduction in the antioxidant activities of the flesh by thermal drying. As the drying temperature increased, the antioxidant activities of the flesh gradually decreased, and they exhibited the lowest level at 90 °C. In contrast to the flesh, the antioxidant activities of the peel gradually increased as the drying temperature increased. Compared to the freeze-dried samples (100%), the antioxidant activity of the 90 °C-dried samples decreased to about 22% in the flesh, while it was increased to about 219% in the peel. Consequently, the 90 °C-dried flesh and peel showed similar antioxidant activity levels, whereas the freeze-dried samples showed over a 6-time difference between the antioxidant activities in the flesh and the peel.

### 3.7. Correlation between Phytochemicals and Antioxidant Activities

In the flesh, all of the phytochemicals showed strong positive correlations with the DPPH, ABTS, and FRAP activities (Table 2). Otherwise, the peel showed a different correlation significance for each phytochemical. The total carotenoids and condensed tannins by a vanillin assay shows significantly negative correlations with the antioxidant activities. No significant correlations against the antioxidant activities were observed in the TPA, in the hydrolysable tannins by a KIO_3_ assay, and in the TF content. The antioxidant activities in the peel showed significantly positive correlations with the hydrolysable tannins and TPs. The correlation values (*r*^2^) between the antioxidant activities and the hydrolysable tannins were 0.9072, 0.9082, and 0.9711 with the DPPH, ABTS, and FRAP, respectively. Moreover, the values between the antioxidant activities and the TPs were 0.9418, 0.9183, and 0.9878 with the DPPH, ABTS, and FRAP, respectively.

## 4. Discussion

The heat processing of fruits causes many morphological changes that accompany the browning of the tissues. Browning was observed only in the 90 °C-dried persimmon flesh and peel. The browning of fruits during processing can involve enzymatic or nonenzymatic browning. The enzymatic activity is strongly related to the water content of the medium [30]. The optimum temperature of polyphenol oxidase, which is the main enzyme in browning, is known to range from 35–40 °C [31]. Because the high temperature of 90 °C denatures enzymes, strong browning at 90 °C is considered to be a nonenzymatic browning reaction, in which the proteins and sugars react with each other (Maillard reaction), or the sugars are thermally decomposed (caramelization reaction) [32]. The high L* values and low a* values of the 60 °C-dried samples indicate that browning hardly occurred. This is because the enzyme is inactivated because of rapid dehydration at 60 °C, and the temperature is relatively low for nonenzymatic browning.

Carotenoids also contribute to the color of the persimmon fruit. In our results, the thermal drying caused carotenoid degradation in both the flesh and peel, as has been previously reported [33,34]. These results suggest that the decrease in the a* value of the 60 °C-dried samples was a color loss that was due not only to the inhibition of the browning, but also to the degradation of the carotenoids. In addition, carotenoids can be isomerized as well as degraded by heat treatment, which causes a color reduction [35]. The heat-induced reduction of the carotenoids and the conversion to various isomer-forms were found in persimmon, tomato, and spinach [11,36]. Isomer-forms of carotenoid have lower bioavailability than trans-carotenoids [37]. The heat-induced degradation and isomerization of carotenoids can also result in the loss of antioxidant activity [11]. In the flesh, our results also show that a decrease in the antioxidant activity was observed, along with a decrease in the carotenoids, depending on the heat intensity. In our results, the carotenoids were mostly present in the ester form, even after the saponification of the dried persimmons. A previous report by Mertz et al. (2009) [38] demonstrates that the antioxidant activity of the ester form was lower than that of the free carotenoid. The antioxidant activity of the flesh had a high positive correlation with the carotenoids. Nevertheless, the flesh with a lower carotenoid content had higher antioxidant activity than the peel. Moreover, the activity of the peel had a negative correlation with the carotenoids. Therefore, our results imply that carotenoids are not a representative antioxidant of persimmon fruit.

The results show that the thermal changes in the tannin contents of the flesh and the peel had different patterns. Both the TCT and THT contents in the flesh were higher than those of the carotenoids and were significantly reduced by heat, exhibiting a strongly positive correlation with the antioxidant activity. Therefore, it could be concluded that the decrease in the TCT and THT contents in the flesh that were caused by thermal processing is the main cause of the decrease in the antioxidant activity. It has previously been reported that condensed tannins with high-molecular weights are the major antioxidants in persimmon flesh [12]. Our results reveal that hydrolysable tannins also contributed significantly to the antioxidant activity in the flesh. In the flesh, the hydrolysable tannins with low-molecular weights (gallic and tannic acids) that were detected by HPLC were increased by heat. Tannins with high-molecular weights are hardly detectable by HPLC [12,14,29], but these can be detected indirectly by a colorimetric assay. The colorimetric analysis and the HPLC results demonstrate that the decrease in the antioxidant activity of the persimmon flesh by heat was due to the decrease in the tannins with higher molecular weights.

Similar to our results on the antioxidant changes, previous studies report that the antioxidant activity was decreased in the flesh of persimmon and increased in the peel by thermal processing, although the authors did not find the reason for the changes in the active molecules [17,26]. We found that the increase was due to the increases in the TP and THT contents. An increase in the fruit antioxidant activity after heat treatment has been frequently reported. This increase can be explained by the increase in the free phenolics that is caused by the cleavage of the phenolics that are covalently bound to the cell-wall components or insoluble polymers [39]. Heat treatment can either degrade bound phenolics (insoluble, nonextractable, or with aqueous organic solvent) into free phenolics (soluble, extractable), or it can further degrade the free phenolics [39]. Meanwhile, dehydration converts the free phenolics into bound phenolics [14]. Persimmon peel has a lower water content compared with the flesh, which reveals that the conversion of the free phenolics to bound phenolics by dehydration hardly occurred. The HPLC results show the increase in the gallic and tannic acid contents of the peel that were caused by heat (Figure 4). These results demonstrate that this increase in the antioxidant activity in the peel by heat was due to an increase in the free phenolics (gallic and tannic acids).

The antioxidant activity of the flesh was also significantly correlated with the TP and TF contents. The TP content of the peel was increased by heat, which shows its positive correlation with the antioxidant activity, whereas the TF content had no significant difference between the drying temperatures. The TP content, including the condensed and hydrolysable tannins, increased, despite the decrease in the TCT in the peel. Therefore, an increase in the TP in the peel could be represented by an increase in the THT. Flavonoids, such as myricitrin, kaempferol, and quercetin, have been previously studied as potential antioxidants in persimmon peel [6,7]. However, there was no correlation between the thermal changes in the antioxidant activity and the TF content, which suggests that flavonoids are not the main antioxidants in persimmon peel. The correlation analysis results clearly show the different thermal changes in the antioxidant activities and phytochemicals in the persimmon flesh and peel. This study can be used as a model for the application of the processing method of persimmon fruit and for the development of products by providing information about the thermal changes in the phytochemicals and the representative antioxidants in each part of the fruit.

## 5. Conclusions

This study proves that the application of the thermal-drying processing of persimmon peel is a useful processing method to improve the hydrolysable tannin and antioxidant activity in the development of the nutraceutical source. Otherwise, thermal drying was found to have a negative effect on the active compounds and on the antioxidant effect of persimmon flesh, and it decreased both the condensed and the hydrolysable tannins. In contrast to the results of the flesh, the hydrolysable tannins and phenolic compounds in the peel were increased by the thermal-drying processing, and they showed a significantly positive correlation with the antioxidant activities. It is assumed that the compounds are the major antioxidant contributors in the peel of astringent persimmon. Although the carotenoids were decreased by the thermal-drying processing in both the flesh and peel, the carotenoid reduction and the increase in the antioxidant activity in the processed peel suggest that carotenoids are not major antioxidant contributors in astringent persimmon fruit.

## Figures and Tables

**Table 1:** Relative contents of carotenoids in the thermally processed persimmons.

Carotenoids	Retention Time (min)	λ Max (nm)	Carotenoid Contents (%) Based on Total Carotenoids of Freeze-Dried (FD) Peel							
			Flesh				Peel			
			FD	30 °C	60 °C	90 °C	FD	30 °C	60 °C	90 °C
Zeaxanthin	12.40	453.1/479.4	0.35 ± 0.04 a	0.30 ± 0.03 a	0.31 ± 0.04 a	0.15 ± 0.02 b	0.76 ± 0.05 b	1.14 ± 0.09 a	0.68 ± 0.02 b	0.38 ± 0.03 c
β-cryptoxanthin	15.43	453.9/480.0	0.96 ± 0.12 a	0.87 ± 0.05 ab	0.67 ± 0.10 b	0.26 ± 0.04 c	3.11 ± 0.05 a	2.86 ± 0.28 a	1.44 ± 0.08 b	0.61 ± 0.04 c
β-carotene	18.85	454.9/480.6	6.19 ± 0.55 a	4.64 ± 0.46 b	0.92 ± 0.07 c	0.34 ± 0.05 c	17.87 ± 0.25 a	11.81 ± 0.88 b	3.81 ± 0.44 c	0.45 ± 0.01 d
β-cryptoxanthin ester	23.48	452.0/480.8	6.06 ± 0.56 a	4.35 ± 0.30 b	0.72 ± 0.13 c	0.27 ± 0.04 c	17.32 ± 0.07 a	10.89 ± 1.05 b	2.2 ± 0.16 c	0.78 ± 0.13 c
β-cryptoxanthin ester	24.20	454.1/481.7	15.94 ± 1.63 a	11.97 ± 0.71 b	1.86 ± 0.21 c	0.62 ± 0.04 c	42.34 ± 0.37 a	28.18 ± 2.84 b	6.13 ± 0.25 c	2.84 ± 0.35 c
Zeaxanthin ester	26.15	449.1/478.2	1.98 ± 0.20 a	1.14 ± 0.04 b	0.14 ± 0.05 c	0.07 ± 0.03 c	3.75 ± 0.36 a	2.3 ± 0.22 b	0.26 ± 0.03 c	0.10 ± 0.02 c
Zeaxanthin ester	26.89	450.3/479.4	7.40 ± 1.09 a	5.77 ± 0.36 a	0.79 ± 0.16 b	0.17 ± 0.04 b	14.06 ± 0.05 a	9.78 ± 0.82 b	1.55 ± 0.18 c	0.56 ± 0.06 c
Zeaxanthin ester	27.49	447.3/469.7	0.64 ± 0.03 a	0.66 ± 0.08 a	0.10 ± 0.04 b	0.10 ± 0.06 b	0.79 ± 0.04 a	0.92 ± 0.1 a	0.43 ± 0.04 b	0.45 ± 0.06 b
Total carotenoids			39.52 ± 4.02 a	29.70 ± 1.95 b	5.49 ± 0.69 c	1.97 ± 0.10 c	100.00 ± 0.00 a	67.87 ± 6.17 b	16.51 ± 0.91 c	6.17 ± 0.57 d

Values are averages with standard errors from triplicate experiments. Different letters within the same line in each flesh and peel indicate significant differences at *p* < 0.05 (Tukey’s HSD test).

**Table 2:** Correlation coefficients between antioxidant activities and phytochemicals in the processed (FD, 30, 60, and 90 °C) persimmon fruit.

		Total Carotenoids	Condensed Tannins		Hydrolysable Tannins		TPC ^2^	TFC ^3^
			Vanillin	TPA ^1^	KIO_3_	FeCl_3_		
**Flesh**	DPPH	0.9211 ***	0.8530 ***	0.9256 ***	0.9799 ***	0.9834 ***	0.9910 ***	0.9562 ***
	ABTS	0.9487 ***	0.8908 ***	0.9400 ***	0.9464 ***	0.9557 ***	0.9595 ***	0.8848 ***
	FRAP	0.9563 ***	0.8863 ***	0.9205 ***	0.9531 ***	0.9722 ***	0.9800 ***	0.9096 ***
**Peel**	DPPH	−0.8394 ***	−0.8980 ***	0.1145 ^ns^	0.2440 ^ns^	0.9072 ***	0.9418 ***	0.2238 ^ns^
	ABTS	−0.7450 **	−0.8385 ***	0.0944 ^ns^	0.4159 ^ns^	0.9082 ***	0.9183 ***	0.0631 ^ns^
	FRAP	−0.7873 **	−0.8391 ***	−0.0628 ^ns^	0.3337 ^ns^	0.9711 ***	0.9878 ***	0.0968 ^ns^

^1^ TPA: total proanthocyanins; ^2^ TPC: total phenolic content; ^3^ TFC: total flavonoid content. Asterisks indicate significance (^ns^ no significance; ** *p* < 0.01; *** *p* < 0.001) by Pearson’s correlation analysis.

## Data Availability

All of the data is contained within the article.
